# Molecular Basis of α-Thalassemia in Iran

**DOI:** 10.22034/ibj.22.1.6

**Published:** 2018-01

**Authors:** Atefeh Valaei, Morteza Karimipoor, Alireza Kordafshari, Sirous Zeinali

**Affiliations:** 1 Department of Molecular Medicine, Biotechnology Research Center, Pasteur Institute of Iran, Tehran, Iran; 2 Medical Genetics Lab of Dr. Zeinali, Kawsar Human Genetics Research Center, Tehran, Iran

**Keywords:** α-Thalassemia, Point mutation, HbH disease, Iran

## Abstract

Alpha-thalassemia (α-thal) is probably the most prevalent monogenic condition in the world. Deletions are the most common types of mutations in α-thal, followed by point mutations and small insertion/deletion. In the context of national screening program for prevention of thalassemia and hemoglobinopathies in Iran, α-thal carriers have come to more attention. Therefore, the frequency and distribution of α-globin mutations in various regions of the country have been studied in recent years. A comprehensive search was performed in PubMed, Scopus, and national databases for finding reports on mutation detection in α-thal carriers and HbH disease with Iranian origin. The mutation data of 10849 α-thal carriers showed that -α^3.7^ and α^-5NT^ were the most common deletional and nondeletional mutations, respectively. In HbH disease cases, the -α^3.7^/--^MED^ was the most prevalent genotype. Overall, 42 different mutations have been identified in α-globin cluster reflecting the high heterogeneity of the mutations in Iranian populations.

## INTRODUCTION

Alpha-thalassemia (α-thal; OMIM: #604131) appears to be the most common single-gene disorder worldwide. It is mostly caused by deletion and less frequently by point mutations in α-globin genes[1,2]. The mutations result in the absence or reduced production of α-globin chains in hemoglobin tetramer.

α-thal is the most frequent disorder in Southeast Asia, but it is also prevalent in the Mediterranean Basin, the Middle East, India, and Sub-Saharan Africa[3,4]. The prevalence of carriers varies between 1% and 98% throughout the tropics and subtropics[5]. α-thal is mainly prevalent in malaria-endemic regions and is hypothesized to protect the individuals from severe form of malaria and its clinical complications[6,7].

There are two copies of α-globin genes (α2 and α1) on each short arm of chromosome 16 (16p33), which makes the pathology of α-thal complicated[8]. α-globin chains in combination with ε-, γ-, δ-, and β-globin chains produce Hb Gower-2 (α2ε2), HBF (α2γ2), HbA2 (α2δ2), and HbA (α2β2), respectively. The majority of α-thal mutations are deletions, and point mutations are found less frequently[9].

In general, α-globin variants result from deletional and nondeletional (ND) mutations in α_1-_ or α_2-_-globin genes, leading to abnormal α-globin chains[10]. There are two main classes of α-thal mutations: α^+^-thal in which only one of the α-globin genes is lost or inactivated, and α^°^ alleles in which two α-globin genes are deleted (in *cis* or *trans*)[11]. A large number of mutations identified in α-globin genes are usually asymptomatic in heterozygous carriers and are detected in routine blood count, but their coinheritance can result in a wide range of hematological and clinical manifestations. Four clinical conditions have already been reported for α-thal:

(A) Individuals with only one α-globin defect (i.e. -α/αα or α^ND^α/αα) are usually asymptomatic with a mild reduction in mean corpuscular volume (MCV) and mean corpuscular hemoglobin (MCH). In α^+^-thal heterozygotes, the indices may overlap with normal range (silent carriers).

(B) Deletion of two α-globin genes (i.e. -α/-α or --/αα) and some ND mutations is usually asymptomatic with severe microcytosis and hypochromia.

(C) Deletion or dysfunction of three α-globin genes (-α/-- or --/α^ND^α) causes hemoglobin H (HbH) disease. The disease is represented by mild to moderate hemolytic anemia, hepatosplenomegaly, jaundice, and gallstone[12-14]; HbH disease that results from deletional forms often shows a mild course of the disease and usually does not need blood transfusion[15]. The ND forms in combination with large deletions are characterized by severe anemia and clinical complications such as splenomegaly, gallstone, leg ulcers, infections, and thrombosis. Less common form of HbH disease has been reported to carry two point mutations (α^ND^α/α^ND^α) in α-globin genes. In HBH disease, a wide range of clinical manifestations has been observed, even in the presence of similar genotype, which is probably due to genetic and environmental modifiers[16].

(D) Complete absence of α-globin genes (--/--) results in hemoglobin Bart’s hydrops fetalis, which is characterized by severe intrauterine anemia and death[17].

Hematological parameters of α-thal trait include MCV <80 fl and MCH <27 pg and HbA2 (<3.5%) and HbF (<2%) levels[18,19]. The most common causes of deletional α^+^ -thal is rightward 3.7-kb (-α^3.7^) and leftward 4.2-kb (-α^4.2^) deletions. Mediterranean (--^MED^) and Southeast Asian deletions (--^SEA^) are the frequent causes of α^º^-thal in the world[20]. More than 750 different variants in α-globin genes have been reported to cause α-thal worldwide[21]. In addition to α-globin gene deletion, the extra copies of α-globin genes (triplication/quadruplication) have been introduced[22]. The excess of α-globin chains is proposed to be caused by misalignment of homologous sequences on the α2 and α1 genes during recombination from unequal cross-over. This situation by itself does not affect the hematological parameters, and carrier individuals are usually asymptomatic. The co-inheritance of this situation with β-thal alleles exacerbates α/non-α-globin chain imbalance in β-thal carriers and may produce β-thal intermedia with a wide range of clinical manifestations[23].

Molecular analysis of α-globin gene rearrangements usually starts with multiplex Gap-PCR for common α-globin gene cluster deletions, including common -α^3.7^, -α^4.2^, -α^2.5^, and --^MED^ deletions[24]. Multiplex ligation-dependent probe amplification (MLPA) technique has been employed for finding unknown deletions/ duplications in α-globin cluster. This method is robust and has superseded Southern blotting and real-time PCR methods[25].

Sanger sequencing, PCR followed by digestion of amplified fragment, and amplification refractory mutation system PCR (ARMS-PCR) methods have been used for detection of ND mutations in carriers of α-thal. Reverse dot blot (RDB) assay has also been exploited for finding common deletions and point mutations in α-globin genes[26]. However, other investigations have used array comparative genomic hybridization (array-CGH) for detecting copy number variations in order to improve the diagnosis of rearrangements in α-thal carriers or HbH disease patients[27].

Iran, a country in the Middle East of Asia with 1,648,000 square km of land and around 80 million populations, is located on thalassemia belt and has a large number of thalassemia patients similar to many other countries in the region. Thalassemias are more prevalent in the Northern (Caspian Sea) and Southern (Persian Gulf and Oman Sea) regions of the country, where the carrier rate for β-thal is ~10%, and α-thal exceeds 35%[28]. Since the Iranian population is a mixture of different ethnic groups, and also α-thal could be misdiagnosed as β-thal, there is an urgent need for the determination of frequency and distribution of α-globin variants in various regions of the country.

Here, we surveyed the studies performed on the distribution and frequency of HBA2 and HBA1 genes, as well as deletional and ND mutations in the carriers of α-thal, in Iran. The present study also includes the reports on the mutation spectrum of HbH disease and genotype-phenotype correlation. Finally, α-triplication in normal individuals and its combination with HBB mutations, and the consequent phenotypes were interrogated.

## METHODS

PubMed and Scopus electronic databases were searched for published reports on mutations in α-thal carriers, HbH disease, and α-triplication in Iran. Original articles and case series studies were retrieved. In addition, for finding the Persian articles, national databases, including the Scientific Information Database (SID), Medlib, and Magiran, were also searched.

The duplicate Persian papers, i.e. those have been published partly in Persian and fully in English journals, were excluded. Genotype-phenotype correlation was retrieved in HbH disease. Based on the above strategy, the data for 10,849 cases with known α-globin gene status were extracted from 13,773 individuals who claimed to be α-thal carrier, from January 2003 to early 2017.

## RESULTS

In total, 37 published articles were selected for analysis; 23 articles on mutation detection in α-thal carriers/cord blood samples and 4 articles and 5 case reports on HbH disease. In addition, 3 original articles and 2 case reports were found on α-triplication in normal individuals or in co-inheritance with β-thal alleles. The obtained reports were categorized according to geographic distributions and ethnic background. Forty-two different mutations were found in α-globin genes that included deletion, duplication, point mutation, and hemoglobin variants[29-47]. Frequencies of mutations and their geographical distribution are shown in Table 1. The most frequent mutation was a 3.7-kb deletion (-α^3.7^) contributing up to 67.1% (n = 8321) of all known reported α-globin alleles (n = 10849). The frequency of -α^4.2^, --^MED^, and -α^20.5^ deletions were 4.68%, 3.6%, and 0.97%, respectively. Among ND mutations, the most common alleles were c.95+2_95+6delTGAGG (α^5NT^α/αα), PolyA2 (AATAAA>AATGAA), Hb Constant Spring (Hb CS), CD19 (-G), PolyA1 (AATAAA>AATAAG), and CD59 (Hb Adana) representing 6.99% (n = 867), 6.78% (n = 845), 3.4% (n = 429), 2.6% (n = 326), 2.37% (n = 296), and 0.25% (n = 32) of mutated α-globin genes, respectively. The -5NT mutation was highly frequent in the southern areas than north of the country[41,45,47], but in the north of Iran, α^polyA2^ was more frequent[34].

**Table 1:** The frequency of mutations causing α-thalassemia in Iranian population

Province/origin	-α3.7 (%)	-α4.2	-α20.5	--^MED^	CD59	5nt	PolyA1	PolyA2	Hb CS	CD19	ND	Rare alleles	Ref
Mazandaran (north)	440 (57.1)	57 (7.4)	0	54 (7.0)1-	0	43 (5.58-)	13 (1.6-)	131 (17.01-)	32 (4.15-)	0	13 (1.6-)	0	29
Gilan (north)	48 (42.5-)	5 (4.5-)	2 (1.77-)	10 (8.84-)	1 (0.9-)	8 (7.07-)	4 (3.5-)	14 (12.4-)	12 (10.6-)	1 (0.9-)	9 (7.9-)	8 (7.07-)	30
Mazandaran (north)	123 (45.2-)	25 (9.1-)	6 (2.2)%	12 (4.4-)	5 (1.8-)	18 (6.6-)	4 (1.5-)	50 (18.3-)	8 (3-)	11 (4.04-)	28 (10.3-)	10 (3.6-)	31
Mazandaran (north)	121 (52.8-)	11 (4.8-)	0	26 (11).3-	0	10 (4.3-)	8 (3..5-)	30 (13.1-)	15 (6.5-)	2 (0.87-)	37 (13.85-)	6 (2.6-)	32
Mazandaran (north)	55 (88.7-)	5 (8.06-)	0	0	0	0	0	0	0	0	80 (56.3-)	2 (3.2-)	33
Mazandaran, Gilan (north)	591 (53.7-)	80 (7.2-)	6 (0.54-)	89 (8.09-)	0	48 (4.3-)	21 (1.9-)	198 (18.01-)	60 (5.4-)	0	97 (15.2-)	6 (0.54-)	34
Tabriz (northwest)	573 (81.5-)	37 (5.2-)	31 (4.4-)	36 (5.1-)	0	12 (1.7-)	3 (0.42-)	8 (1.1-)	0	0	606 (-86.2-)	3 (0.42-)	35
Tehran*	100 (57.8-)	7 (4.04-)	10 (5.8-)	14 (8.09-)	0	25 (14.4-)	1 (0.57)	7 (4.04-)	6 (3.5-)	3 (1.7-)	14 (8.09-)	0	36
Tehran (referral center)*	395 (60.4-)	46 (7-)	12 (1.8-)	30 (4.6-)	8 (1.2-)	33 (5-)	11 (1.7-)	67 (10.2-)	22 (3.3-)	15 (2.3 (-	3 (0.45-)	14 (2.1-)	37
Esfahan (center)	219 (70.8-)	15 (4.86-)	12 (3.9-)	3 (0.9)7-	0	27 (8.7-)	5 (1.6-)	13 (4.2-)	9 (2.9-)	5 (1.6-)	27 (8.03-)	1 (0.32)	38
Hormozgan (south)	91 (79.1-)	2 (1.7-)	0	0	0	5 (4.3-)	0	0	0	14 (12.1-)	7 (5.7-)	3 (2.6-)	39
Kohgiluyeh and Boyer-Ahmad (south)	857 (71.3-)	44 (3.7-)	6 (0.5-)	23 1.9-	0	84 (6.7-)	50 (4.1-)	47 (3.9-)	45 (3.7-)	21 (1.7-)	805 (40.1-)	24 (2-)	40
Shiraz (south)	3185 (68.6-)	177 (3.8-)	35 (0.75-)	99 (2.1-)	0	383 (8.2-)	141 (3.03-)	195 (4.2-)	217 (4.6-)	182 (3.9-)	1453 (23.8-)	25 (0.5-)	41
Khozestan (southwest)	70 (84.3-)	2 (2.46-)	0	6 (7.2-)	0	4 (4.8-)	0	1 (1.2-)	0	0	14 (14.4-)	0	42
Khozestan (southwest)	69 (60.5-)	8 (7-)	0	9 (7.9-)	1 (0.87-)	5 (4.4-)	2 (1.75-)	9 (7.9-)	1 (0.87-)	5 (4.4-)	12 (9.5-)	5 (4.4-)	43
Khozestan (southwest)	102 (70.8-)	2 (1.4-)	1 (0.7-)	14 (9.8-)	0	3 (2.1-)	4 (2.8-)	1 (0.7-)	2 (1.4-)	12 (8.4-)	2 (1.37-)	2 (1.4-)	44
Sistan and Baluchestan (southeast)	245 (76.5-)	8 (2.5-)	0	1 (0.3-)	0	53 (16.5-)	0	0	0	13 (4.06-)	954 (74.8-)	0	45
Kerman (southeast)	617 (83.8-)	27 (3.6-)	0	2 (0.2)7	0	31 (4.2-)	4 (0.54-)	4 (0.54-)	0	42 (5.7-)	24 (3.1-)	9 (1.2-)	46
Kermanshah (west)	470 (67-)	23 (3.27-)	0	22 (3.1-)	17 (2.4-)	75 (10.6-)	25 (3.5-)	70 (9.9)7-	0	0	45 (6.02-)	0	47

Rare mutations: CD14, IVSI-138, IVSII-148, CD22, CD99, CD108, IVSI-4, Int.codon, anti 3.7, CD26, CD130, CD12, IVSI-116, IVSII-55, 3UTR, CD58, CD24, CD142, CD124, -α^ST^, CD103, CD21, CD16, THAL, FIL, Icaria,CD93-98, ND: not determined;

* Samples from different regions of the country

Regarding HbH disease, four reports were eligible to be included in the study[48-51]. The genotype and clinical parameters of the patients are summarized in Table 2. As illustrated in the Table, the α-globin genotypes of 168 Iranian patients affected with HbH disease. The most common genotype in all studies, which was found in 52 (30.9%) patients, was --^MED^ */*-α^3.7^, followed by -α^20.5^/-α^3.7^, --^MED^ /CS, -α^20.5^/-α^5NT^, and --^MED^ /α^polyA2^α with 11.9% (n = 20), 7.1% (n = 12), 7.1 % (n = 12), and 5.3% (n = 9), respectively.

**Table 2:** Genotypes in Iranian HbH disease patients

Mutation	Number (%)	Number (%)	Number (%)	Number (%)
3.7/20.5	11 (22.4)	-	4 (10)	5 (8)
--^MED^/3.7	17 (34.6)	4 (30.7)	10 (25)	21 (32)
--^MED^/C.S	3 (6.1)	2 (15.3)	3 (7.5)	4 (6)
3.7/unknown	3 (6.1)			
20.5/C.S	1 (2.04)		1 (2.5)	3 (4.5)
--^MED^/5nt	1 (2.04)		1 (2.5)	1 (1.5)
--^MED^/PolyA2	5 (10.2)		2 (5)	2 (3)
20.5/PolyA2	3 (6.1)		2 (5)	3 (4.5)
--^MED^ /21 bp del	1 (2.04)		2 (5)	2 (3)
--^MED^/IVSII+4-21ntlNS	1 (2.04-)--			
20.5/CD59	2 (4.08)			
20.5/CD99	1 (2.04)		1 (1.5)	
5nt/20.5			6 (15)	6 (9)
3.7/C.S		1 (7.69)		
3.7/T-Saudi		2 (15.3)		
--^MED^/T-Turkish		1 (7.69)		
T-Saudi Homo		2 (15.3)		
--^MED^/unknown		1 (7.69)		
--^MED^/4.2			1 (2.5)	1 (1.5)
3.7/CD19			1 (2.5)	
PolyA2/PolyA2			3 (7.5)	
PolyA1/PolyA1			3 (7.5)	6 (9)
C.S/C.S			1 (2.5)	1 (1.5)
--^MED^/PolyA1				3 (4.5)
3.7/CD19				3 (4.5)
CD59/--^MED^				1 (1.5)
--^MED^/CD19				1 (1.5)
3.7/PolyA1				1 (1.5)
α^Sun prairie^/α^Sun prairie^				1 (1.5)
Ref	48	49	50	51

The data of extra-globin genes were considered in the present study. In a study published by our group, the α-globin gene triplication was tested in 280 individuals with Iranian origin using Gap-PCR, and positive cases were confirmed by MLPA method. The data showed that 6 (2.14%) individuals had ααα^anti3.7^ in heterozygous form[52]. In another study in the north of Iran, Mazandaran Province, Jalali *et al*.[53] has investigated the frequency of α-globin gene triplication in neonates and determined ααα^anti3.7^ in 9 out of 412 (2.1%) of those cases. The remaining studies on α-globin triplication have been performed in β-thal intermedia (β-TI) and β-thal major (β-TM) patients who often carry one mutation in HBB gene in heterozygous form). Farashi *et al*.[54] have reported the co-inheritance of ααα^anti3.7^ in 14 β-TI and 9 β-TM patients. Among β-TI patients, three cases were homozygote or compound heterozygote of β-thal alleles, whereas in β-TM group, two patients had heterozygote β-thal allele. All patients were heterozygous for the ααα^anti 3.7^ triplication. In two case reports, the co-inheritance of IVSI-5 mutation in HBB gene, in heterozygote state with ααα^anti3.7,^ has been studied. The co-inheritance of α-globin triplication with β-thal allele resulted in β-TI[55,56].

## DISCUSSION

Thalassemia and hemoglobinopathies are common hereditary disorders worldwide that impose a growing burden on global health. The main clinical manifestations of these disorders are hemolytic anemia with different severities. Screening program of β-thal has been conducted in some countries around the world including Iran in the Middle East region[57]. The national program for the prevention of thalassemia in Iran has been started since 1997 in whole country. In this program, more than one million partners are screened for thalassemia carrier status at premarital stage annually. The program has been conducted with the aim of screening β-thal carriers and preventing the birth of β-thal major[58,59]. Although the extended support for screening α-thal carriers and genetic counseling has been provided, no independent screening for α-thal has been performed in the country.

The referral of families for genetic counseling and molecular characterization in the context of the program shows that the rate of individuals who have reduced red blood cell indices with normal HbA2 and HbF has been increasing in the program. The main differential diagnosis of these cases is usually iron deficiency anemia, α-thal carrier, and normal HbA2 β-thal carrier. Hence, these individuals are usually remaining in the screening program for further characterization and discrimination with silent β-thal carriers. Therefore, in recent years, more attention has been paid to α-thal and its clinical significance. In last decade, different studies have been performed on the molecular basis of α- and β-thal in different regions of the country, though on a limited number of samples. The data from studies have usually been obtained from molecular analysis of individuals referred to genetic laboratories in the context of screening program of β-thal.

No comprehensive study has been performed regarding the prevalence of α-thal carriers in different regions in Iran. At least two reports were found to investigate the prevalence of carriers in north and south of the country. Harteveld *et al*.[39] reported the elevated levels of Hb Bart’s in 218 out of 618 (33%) randomly collected blood samples in Hormozgan Province, south of Iran. However, not all α-thal carriers showed the elevated levels of Hb Bart’s, and the prevalence of the α-thal carriers might be underestimated. Jalali *et al*.[53] investigated 412 neonates in Mazandaran Province, north of Iran, by multiplex Gap-PCR for detection of three common deletions (-α^3.7^, -α^4.2^, and --^MED^) and -5NT mutation by PCR-RFLP. Among 412 neonates, they found at least one deletion or point mutation in 56 cases (13.5%). It should be noted that the frequency of the α-thal carriers should be higher in this region because in this report, only four common mutations has been investigated in cord blood samples. In another study by the same group in Mazandaran, Sari, positive Hb Bart’s was found in 69 out of 680 (10.1%) cases, in a single center study from 2007 to 2008[60].

In this study, we surveyed the molecular and genotype-phenotype correlation data of reports on molecular analysis of α-thal in different regions and provinces of the country, published till the early 2017. The data of 10849 α-thal carriers with known genotype were included in this study (Table 1). The data shows that the -α^3.7^ mutation is the most common deletion causing α-thal in Iran. The mutation is more common in northwest[35], center[38], south[39-41], west[47], southwest[42-44] and southeast[45,46] than north and northeast of Iran[29-32,34]. The overall frequency of *cis* deletional forms of α-thal --^MED^ and -α^20.5^ kb) in this study was 4.5%. None of the common large deletions in South East Asia, i.e. SEA, FIL, and THAI, was identified in the Iranian population except in two cases with THAI and FIL large deletions from north of Iran[33]. In addition, unknown large deletions have been detected by some studies that have exploited real-time PCR[61] and MLPA[62] methods.

The -5NT and α^polyA2^ (AATAAA>AATGAA) alleles are the most common point mutations. The frequency of α^polyA2^ in α-thal carriers is decreasing from north[34] to central and south of the country, whereas the -5NT mutation has been reported with higher frequency in south[41], southeast[45], and west[47] of the country than north.

A point mutation that has frequently been reported in the country, especially in Northern Provinces, is Hb CS. The mutation was originally found in Southeast Asia and Southern China. Later, it was detected in Mediterranean area with different haplotypes[63]. Hb CS is an unstable Hb variant that results from mutation in the stop codon (HBA2: c.427T>C, codon 142 TAA>CAA). The loss of stop codon produces an elongated unstable mRNA. The frequency of Hb CS has been reached to more than 10% of known α-thal alleles in Gilan Province[30].

In spite of PolyA2 mutation, the frequency of PolyA1 allele is highly similar in northern and southern regions. CD19 (-G) mutation has the highest frequency in Hormozgan Province, in the south of the country, with ~12% of detected alleles[39]. The next common point mutation, CD 59 Gly>Asp or known as Hb Adana (HBA2: c.179G>A), has been reported in different regions of the country. The highest frequency was seen in the west of country in a report from Kermanshah Province with 2.4% of the known alleles[47]. Overall, in Iranian population the frequency of deletional alleles in α-thal carriers is significantly higher than ND alleles (76.35% vs. 22.39%). Based on the obtained data, we can predict that the most common clinical form of α-thal in Iran is HbH disease, rather than hydrops fetalis (as seen to be prevalent in the Southeast Asian population). In addition to α-thal traits, we gathered data of four eligible studies on the molecular data and related phenotypes of HbH disease in Iran. In 2011, a paper was published regarding the HbH disease by Lal *et al*.[15], and soon after the HbH disease entry was added to the OMIM site (https://www.ncbi.nlm.nih.gov/omim) to reflect the importance of the disease, and new attention was given to it.

There are some challenges in classification and predicting the clinical outcome of HbH disease patients based on molecular reports by Lal *et al*.[15] and Zeinali *et al*.[48]. However, previous studies subdivided the patients into two transfusion- and non-transfusion dependent groups[48-51]. In the first group, the deletional form (-α^3.7^/--^MED^) is the most common cause of the disease (Table 2). HbH disease can be a result of the combination of a large deletion and point mutation (--/α^ND^). In this group, the combination of --^MED^ deletion with two point mutations (PolyA2 and Hb CS) are the most common cause of transfusion-dependent HbH disease.

Nearly, all HbH disease patients with -α^3.7^/--^MED^ and -α^3.7^/-α^20.5^ genotypes in published reports from Iran do not need blood transfusion. However, in a report from 10 cases with -α^3.7^/--^MED^ genotype, three needed blood transfusion[50]. Therefore, it is expected that the HbH disease patients with -α/-- genotype show a mild clinical course of disease rather than αα^ND^/--, which is in agreement with previous published reports[15,64]. The clinical course of nondeletional HbH disease is often more severe. Nondeletional mutations usually occur at crucial regions of the gene or produce unstable α-globin chain variant lead to the precipitation of hemoglobin in RBC and membrane damage and consequently cause hemolytic anemia. Molecular studies on HbH disease patients have also shown αα^ND^/αα^ND^ genotype in a few percentage of cases with moderate to severe phenotype. α^CS^α/α^CS^α, α^polyA2^α/α^polyA2^α, and α^polyA1^α/α^polyA1^α are well known genotypes and frequently reported[48-51]. In addition, HbH disease patients with the following genotypes have been investigated in some case reports: c.*93_*94delAA/c.*93_*94delAA[65], α^Dartmouth^α/α ^Dartmouth^α[66], αα/α^poly A1^α^21nt^[67], and α^Sun Prairie^α/α^Sun Prairie^ α[51].

Unexpectedly, in HbH patients presented, the -α/α^ND^α genotype has been introduced as the molecular defect causing HbH disease in a few cases. For instance, -α^3.7^/α^codon19^α has been detected as the molecular basis of the disease in one patient with no history of blood transfusion[50] and -α^3.7^/α^polyA1^α genotype in a 36-year-old patient with the history of occasional blood transfusion, splenectomy at age 22, and first transfusion at age 20[51]. Yavarian *et al*.[49] have found α^3.7^/α^T-Saudia^α and α^3.7^/α^CS^α genotypes in two and one HbH disease patients, respectively[49]. At their cohort study, the lowest Hb level (6.7 g/dL) was measured in association with the α^3.7^/α^CS^α genotype.

The genotype-phenotype correlation is not well-characterized, particularly in ND HbH disease. Environmental and genetic modifiers have been investigated in the patients; however, little progress has been made with clinical applications. Iron overload has been proposed as a time-dependent factor that is related to the clinical severity of the HbH disease, even in non-transfused patients[68]. The impact of hemochromatosis (HFE)[68], glucuronosyltransferase family 1 member A1 (UGT1)[69], and α-hemoglobin stabilizing protein (AHSP)[70] variants needs to be clarified on the phenotype of HbH disease. αAHSP is an erythroid-specific protein that forms a stable complex with free α-hemoglobin and protects it from precipitation in RBCs. Free α-hemoglobin is expected to be a complication of β-thal major when the excess of free α-hemoglobin makes α_4_ tetramer.

In summary, the data from molecular studies of α-thal carriers and HbH disease patients in Iran shows a high heterogeneity of alleles. As it is depicted in Tables 1 and 2, 42 different mutations have been detected, from which 28 mutations are rare, and their allele frequency is less than 1%. The data of genotype-phenotype correlation in α-thal may inform families about reproductive risk and the importance of genetic counseling. The combination of different studies on molecular basis of α-thal in Iran shows the high heterogeneity of alleles causing the disease. Although the hydrops fetalis and transfusion-dependent HbH disease are not as common as β-thal major in Iran, the relative high frequency of α-thal carriers is associated with some challenges in genetic counseling of families for prevention of β-thal major. Our data could provide a valuable basis for genetic counseling and prenatal diagnosis of α-thal.
